# *In Vitro* Effect of Aqueous Extract and Fraction IV Portion of *Ximenia americana* Stem Bark on *Trypanosoma congolense* DNA

**DOI:** 10.1155/2014/904318

**Published:** 2014-01-22

**Authors:** Victor Ambrose Maikai, Beatty Viv Maikai, Patricia Ishyaku Kobo

**Affiliations:** ^1^College of Agriculture and Animal Science, Ahmadu Bello University, P.M.B. 2134, Mando, Kaduna, Nigeria; ^2^Department of Public Health and Preventive Medicine, Faculty of Veterinary Medicine, Ahmadu Bello University, Zaria, Nigeria; ^3^Pharmacology and Toxicology Department, Faculty of Veterinary Medicine, Ahmadu Bello University, Zaria, Nigeria

## Abstract

Trypanosomosis is a debilitating disease affecting mainly livestock and humans in tropical Africa. Chemically synthesized drugs and medicinal plants have been used in the treatment and control of this disease. In this study, the *in vitro* effect of aqueous extracts and fraction IV extract of *Ximenia americana* stem bark on *Trypanosoma congolense* DNA was investigated. The extracts were incubated with the parasites *in vitro* at 300 mg/mL aqueous extract and 25 mg/mL fraction IV portion for 30, 60, and 120 mins. The DNA of the trypanosomes was isolated and digested using ECOR1 enzyme and subsequently PCR was carried out. Results showed that aqueous extract and fraction IV portion immobilized 55% and 90% of the trypanosomes after 30-minute incubation. Subsequent isolation of the parasite DNA and agarose gel electrophoresis did not reveal that cell death was as a result of DNA fragmentation. This suggests that cell death was by another mechanism of action.

## 1. Introduction

African animal trypanosomiasis (AAT) is caused by *Trypanosoma congolense, Trypanosoma vivax*, and* Trypanosoma brucei*. Trypanosomiasis is a debilitating disease of man and domestic and wild animals which is often characterized by anemia, reduced productivity, and high mortality [1–3]. It is a major constraint to the development of livestock in Sub-Saharan Africa [2, 4]. The chemotherapy of African trypanosomes is beset with several problems; these include limited repertoire of trypanocides, resistance to drugs, and toxicity to a protracted treatment protocol [5–7]. Decades of attempts to create a vaccine against trypanosomes have failed due to the development of variable surface glycoprotein, a defense mechanism against the immune system [8]. Economic considerations of the pharmaceutical industry outweigh all others, because of the very low return of the developmental costs.

These factors emphasize the need for research into better and cheaper and eco-friendly trypanocides. Plants are an important part of the culture and traditions of Africa as most rural communities in African are highly reliant on medicinal plants for their health care needs; this is due to the accessibility and affordability of these plants [9]. The search for new drugs from natural sources is a widely used approach that has been successful in the detection of compounds for the treatment of some parasitic diseases [10, 11]. Extracts as well as pure compounds obtained from plants have been reported to possess significant antiprotozoan activities with no side effects [12, 13].

*Ximenia americana *is a plant used in traditional medicine for the treatment of malaria, leproutic ulcers, and infectious diseases [14–17]. The plant has been reported to show antimicrobial, antifungal, anticancer, antitrypanosomal, antioxidant, analgesic, and antipyretic properties [17–21]. The crude extracts consist of complex mixture of compounds which include flavonoids, saponins, alkaloids, quinines, terpenoids, glycosides, and steroids [14, 16, 19]. We have previously reported the trypanocidal activity of *Ximenia americana* aqueous extract against the bloodstream form of *Trypanosoma congolense *[20].

The studies were done to determine if the extracts of *Ximenia americana* had any effect on the DNA of *Trypanosoma congolense in vitro*.

## 2. Materials and Methods

### 2.1. Trypanosome (Parasite)

*Trypanosoma congolense* (Federe strain) was obtained from the Nigerian Institute of Trypanosomosis Research, Vom, Plateau State, Nigeria, and passaged into rat which was subsequently maintained by passages in mice.

### 2.2. Extraction of Plant Material

Two hundred (200 gm) grams of the stem bark powder was weighed into a thimble and then transferred into a Soxhlet extractor and extracted sequentially with petroleum ether, methanol, and water. The extracts were individually collected after each extraction and concentrated using a rotary evaporator (Buchi, Switzerland) at 50°C under reduced pressure and then dried. The solvent free extracts were then weighed and stored in brown bottles at 4°C until use.

### 2.3. Partial Purification of Aqueous Crude Extracts (Column Chromatography)

The aqueous crude extract was partially purified using column chromatography. Briefly, slurry was prepared by shaking 120 g of silica gel (Qualikems, 60–120 mesh powder) with 200 mL of water and methanol in the ratio of (1 : 1) and then packed in a column (1.5 × 30) at a flow rate of 0.2 mL/min^−1^. The column was loaded with 20 mL of the aqueous extract that had been previously adsorbed from distilled water on 4 g of the silica gel and then eluted with four solvent mixtures (ethyl acetate/methanol 19 : 1; benzene/methanol 19 : 1; acetic acid/methanol 1 : 1; water/methanol 1 : 1) in order of increasing polarity. A total of 5 mLs of the eluents were collected in separate beakers and dried at 50°C using a water bath. The dried fractions were kept at 4°C for use in *in vitro *experiments. The fractions were tested for antitrypanosomal activity and fraction IV (water/methanol 1 : 1) which had the highest activity was used for the experiment.

### 2.4. Infection

Three apparently healthy rats were infected with *T. congolense* at peak parasitemia (10^8^ parasite/mL of blood), the animals were sacrificed, and blood was immediately collected in heparinized tubes containing three (3) mL of phosphate buffer saline glucose.

### 2.5. *In Vitro* Incubation of Parasites with Extracts

The blood containing parasites was divided into three replicates of three groups; each tube containing 2 mL of blood was incubated with the extract at 37°C in a water bath as follows.

 Group A1: incubated with aqueous extract (300 mg/mL) for 30 minutes.
 Group B1: incubated with fraction IV (25 mg/mL) for 30 minutes.
 Group C1: untreated control group.
 Group A2: incubated with aqueous extract (300 mg/mL) for 1 hour.
 Group B2: incubated with fraction IV (25 mg/mL) for 1 hour.
 Group A3: incubated with aqueous extract (300 mg/mL) for 2 hours.
 Group B3: incubated with fraction IV portion (25 mg/mL) for 2 hours.

At the end of the time intervals, parasites were isolated from each of the groups using DEAE cellulose [22]. The trypanosomes were washed 3 times with PBSG by centrifugation and decantation to concentrate and store at 4°C prior to use.

### 2.6. DNA Extraction from Isolated Treated and Untreated Parasites

The parasite DNA was extracted using ZR genomic DNA kits from Zymo Research South Africa according to the manufacturers recommended protocol.

### 2.7. Restriction Endonuclease Digestion of DNA with EcoR1

After isolation of the DNA from the treatment groups, the Groups A, B, and C were each divided into two portions, to the first portion it was the uncut DNA (Groups A1, B1, and C1), to the second portion (Groups A2, B2, and C2), it was 5 µL portions of the genomic DNA incubated with 5 µL of EcoR1 concentration 10 µ/µL in 10 µL EcoR1 buffer in a water bath at 37°C for 1 hour.

 Group A1: uncut DNA (parasite incubated with aqueous extract).
 Group A2: EcoR1 digested DNA.
 Group B1: uncut DNA (parasite incubated with fraction IV portion).
 Group B2: EcoR1 digested DNA.
 Group C1: uncut DNA (parasite untreated control).
 Group C2: EcoR1 digested DNA.

### 2.8. PCR Amplification of Isolated Parasite Treated DNA

The amplification of the DNA was carried out according to manufacturers protocol (Fermentas); briefly, PCR master mix 2X was gently vortexed after thawing. Then 25 *μ*L of Maxima hot start Green PCR mix 2X was measured by a PCR tube followed by the addition of 0.1 *μ*L forward primer TCS1 (5′dCGAGAACGGGCACTTTGCGA3′) and reverse primer TCS2 (5′dGGACAACAAAGAAATCCCGCACA3′), respectively; this was followed by the addition of 1 *μ*L of DNA template then 23.8 *μ*L of nuclease free water was added to make 50 *μ*L volume. Reaction mixture was gently vortexed and the samples spun down then were loaded into the thermal cycler to undergo amplification according to Masiga et al. [23]. The reaction conditions were as follows: (30 cycles) 94°C for 60 s, 55°C for 120 s, and 72°C for 120 s.

### 2.9. Agarose Gel Electrophoresis

About 0.8 grams of agarose was dissolved by heating in 100 mL 1X Tris acetic acid and ethylenediaminetetraacetic acid (TAE buffer) (pH 7.9) in a microwave oven set for 2 minutes. The gel was taken out and allowed to cool sufficiently to 60°C. After cooling, 2 *μ*L ethidium bromide stock (100 mg ethidium bromide tablet dissolved in 10 mL distilled water and stored in foil-covered bottle to protect from light) was added and mixed with the gel. Care was taken while handling because it is a mutagen. The gel was poured into cassette trays with a comb inserted. Gels were allowed 30 minutes to set. 30 mLs of 1X TAE buffer was added to the tank and the combs were removed by pulling it out straight up. The tank was filled to mark with 1X TAE. Bromophenol tracking blue was used as the loading dye (0.001 gram bromophenol blue in 1 mL distilled water and 9 mL 40% sucrose solution made in 1X TAE). The solution was filtered and kept in a foil-covered bottle. Two (10 *μ*L) of the marker and samples were separately mixed on a paraffin paper with 2 *μ*L loading dye. The first well was loaded with the control DNA, the subsequent wells were loaded with extract treated DNA, and the last well was loaded with 1 Kb molecular marker. Electrophoresis was carried out for 45 minutes at 80 v. Gels were removed and viewed under ultraviolet radiation for resolved bands; the gel was photographed and documented.

## 3. Result

### 3.1. Effect of Incubating Extracts of *Ximenia americana* on Parasites DNA for 30, 60, and 120 Minutes

**Figure 1:**
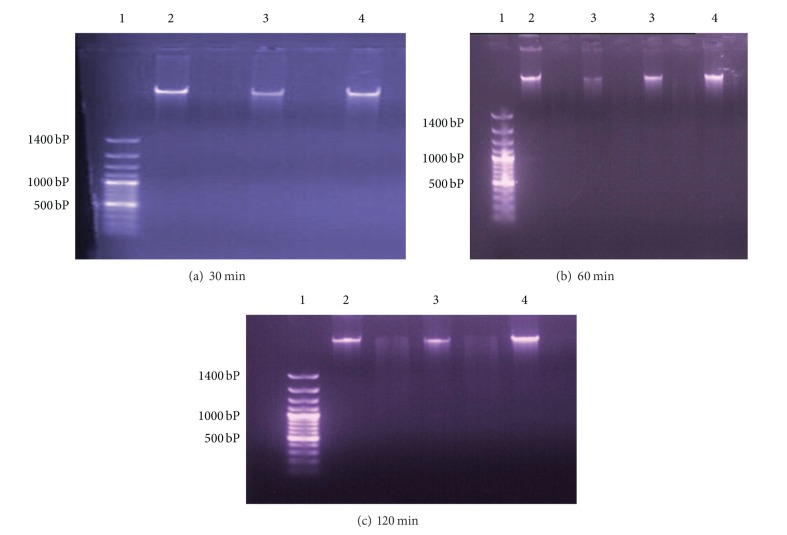
Showing the agarose gel electrophoresis of the isolated DNA of the parasites after incubating the blood with *Ximenia americana* extracts for 30, 60, and 120 mins. Lane 1 DNA marker 100 bp plus; Lane 2, treatment with 300 mg/mL aqueous extract; Lane 3, treatment with 25 mg/mL fraction IV portion; Lane 4, untreated group.

The *in vitro* extract treated trypanosomes with 300 mg/mL aqueous extract and 25 mg/mL fraction IV portion induced 55% and 90% cell death of the parasites after 30-minute incubation, respectively. The DNA of the treated and untreated parasites incubated for 30, 60, and 120 minutes was isolated and electrophoresed and was observed to have high molecular weight bands of >1400 bp (Figures 1(a), 1(b), and 1(c) and Lanes 2, 3, and 4). The bands were detected at the same area with the untreated control group Lane 4.

### 3.2. DNA Amplification of Treated and Untreated Parasite Using PCR

**Figure 2:**
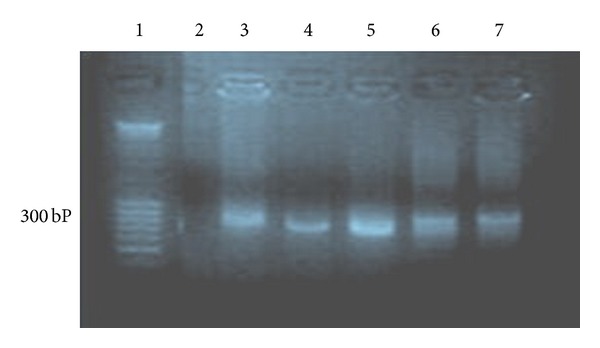
Agarose gel electrophoresis PCR products of the isolated DNA of the parasites after treatment with *Ximenia americana* extract. Lane 1, DNA marker 100 bp plus; Lane 2, water (negative control); Lane 3, untreated group; Lane 4, treatment with 25 mg/mL fraction IV portion; Lane 5, treatment with 3.5 mg/kg body weight diminazene aceturate group; Lane 6, trypanosome genome (Bremen); Lane 7, trypanosome genome (Vom) (positive control).

Amplification of the extract treated and untreated parasite DNA using PCR is shown (Figure 2). The amplification resulted in a specific size band of >300 bp which is specific for *Trypanosoma congolense *for all the groups. There was no indication of DNA fragmentation by the extracts. No fragments below 180 bp were detected for all the treated groups.

## 4. Discussion

The emergence and spread of resistance to antitrypanocidal drugs have highlighted the need for the discovery and development of novel antitrypanocidal leads. One of the approaches used in chemotherapy of parasites relies on testing for biological activity of plant extracts. They offer novel possibilities of obtaining new compounds that could be active against parasites. The results of the *in vitro* incubation of the aqueous extracts and fraction IV portion of *Ximenia americana* showed they had activity against the parasites. The results corroborate those of earlier studies on medicinal plants having antitrypanosomal activity [2, 4, 6, 24, 25]. However, studies of antitrypanosomal activity of plant extracts are mostly limited to testing the ability to inhibit parasite growth *in vitro* and *in vivo*. It is difficult to speculate the mechanism by which the extracts exhibited their trypanocidal action. Phillipson and O'Neil [26] and Atawodi et al. [27] speculated that plant extracts could bind with the kinetoplast DNA of the parasite. Hu et al. [28], Dou and Li [29], Kumar et al. [30], El-Shemy et al. [31], and Saboo et al. [32] have reported that some medicinal plant extracts induced cell death through DNA fragmentation. Hence, we decided to examine if the mode of trypanocidal action was through the binding of the aqueous extracts and fraction IV portion to the DNA of the parasite. Rosenkranz and Wink [33] had earlier reported that plant natural product can interact with molecular target such as DNA and could damage it by fragmentation. DNA fragmentation occurs when endogenous endonucleases or natural products cleave the chromatin DNA into internucleosomal fragments of 180 bp and their multiples [34]. The results of the agarose gel DNA analysis of the *in vitro* extract treated parasites did not reveal any DNA fragments of less than 500 bp, suggesting that death of the parasites was not as a result of DNA fragmentation. The EcoR1 digest of *Trypanosoma* DNA also did not reveal any change in band size of DNA from the untreated group. Our result is similar to [35] who reported that *Azadirachta indica* extracts failed to cause DNA fragmentation in some cancer cells. The result differs from [34] who reported that some plant extracts with trypanocidal effect also fragmented trypanosome DNA causing cell death. The findings of this investigation clearly show that the aqueous extract and fraction IV portion of *Ximenia americana* had antitrypanosomal potentials but did not indicate that trypanosome death was as a result of DNA fragmentation. Further work is on to purify, isolate, and characterize the active principle of fraction IV portion of the extract of *Ximenia americana*.
